# Alzheimer’s disease pathology propagation by exosomes containing toxic amyloid-beta oligomers

**DOI:** 10.1007/s00401-018-1868-1

**Published:** 2018-06-13

**Authors:** Maitrayee Sardar Sinha, Anna Ansell-Schultz, Livia Civitelli, Camilla Hildesjö, Max Larsson, Lars Lannfelt, Martin Ingelsson, Martin Hallbeck

**Affiliations:** 1 Department of Pathology, Department of Clinical and Experimental Medicine, Linköping University, Linköping, Sweden; 2 Department of Public Health and Caring Sciences, Geriatrics, Uppsala University, Uppsala, Sweden; 3 BioArctic AB, Warfvinges väg 25, 112 85 Stockholm, Sweden

**Keywords:** Alzheimer’s disease, Exosomes, Oligomers, Beta-amyloid, Human, Prion-like, Propagation

## Abstract

The gradual deterioration of cognitive functions in Alzheimer’s disease is paralleled by a hierarchical progression of amyloid-beta and tau brain pathology. Recent findings indicate that toxic oligomers of amyloid-beta may cause propagation of pathology in a prion-like manner, although the underlying mechanisms are incompletely understood. Here we show that small extracellular vesicles, exosomes, from Alzheimer patients’ brains contain increased levels of amyloid-beta oligomers and can act as vehicles for the neuron-to-neuron transfer of such toxic species in recipient neurons in culture. Moreover, blocking the formation, secretion or uptake of exosomes was found to reduce both the spread of oligomers and the related toxicity. Taken together, our results imply that exosomes are centrally involved in Alzheimer’s disease and that they could serve as targets for development of new diagnostic and therapeutic principles.

**Electronic supplementary material:**

The online version of this article (10.1007/s00401-018-1868-1) contains supplementary material, which is available to authorized users.

## Introduction

Gradual accumulation of toxic amyloid-beta (Aβ) and tau are believed to be central to Alzheimer´s disease (AD) pathogenesis. These abnormal deposits typically appear with a hierarchical spatial distribution, which suggest that the pathological proteins can propagate between different brain areas [7, 52, 53]. Accordingly, recent studies have demonstrated transfer of Aβ in cellular and animal models [27, 38–40, 61] as well as spread of tau between neurons in the brain of transgenic mouse models [25]. These observations are further supported by the distribution of Aß and tau PET ligands in relation to the brain connectome [45].

Different Aβ conformational states have different properties, and intermediate products of fibril formation, such as lower molecular weight Aβ oligomers (oAβ) and protofibrils, have been suggested to be particularly neurotoxic and to act as seeds for further aggregation [27, 61]. In addition, these soluble forms of Aß correlate better than fibrils with cognitive function in the AD brain [39]. We have previously shown that oAβ can accumulate inside cells and subsequently spread from one cell to another [15, 40]. These findings thus suggest that Aβ can propagate pathology in a manner similar to what is seen for prion disorders and for several other neurodegenerative diseases [8]. However, the underlying mechanisms for the spreading of toxic oAβ remain incompletely understood [28].

Exosomes, small extracellular vesicles (20–120 nm in diameter) [58] developing from endosomes through multivesicular bodies, have recently emerged as key players in cellular communication and transport of molecules in both health and disease, including neuronal toxicity [33] and neurodegenerative disorders [59]. Exosomes can carry different cargos such as proteins, RNA and miRNA and can also contain monomeric Aß, tau and α-synuclein [12, 17, 46, 59] and can propagate tau pathology [4]. Thus, exosomes could potentially also carry aggregated proteins such as oAß. However, no study has so far investigated if exosomes isolated from AD brain tissue can be responsible for interneuronal protein transfer.

For the first time, we now demonstrate that intracellular oAβ is co-localized with exosomes and show that AD brain-derived exosomes can mediate neuron-to-neuron propagation of oAß. Furthermore, we show that the concentration of oAβ in exosomes is significantly increased in post mortem AD brains. In addition, exosomes carrying oAβ can be internalized in cultured neurons and spread their toxic content to nearby cells.

## Methods

### Brain tissues

Post mortem brain samples of temporal neocortex from healthy control (1 female, 4 male) and AD (4 female, 1 male) subjects (Table 1) were provided by the brain bank at Uppsala University, Sweden. The tissue was received as fresh-frozen or as formalin‐fixed (4% formaldehyde) paraffin-embedded blocks. The AD cases were neuropathologically diagnosed as CERAD C, Braak stages IV–VI. None of the control patients suffered from dementia or any neurodegenerative disorder. The collection and use of post mortem brain tissue was approved by the Regional Ethical Committee in Uppsala, Sweden (2005/103, 2005-06-29; 2009/089; 2009-04-22).

**Table 1:** Demographic and clinical characteristics of post mortem cases used in the study

Diagnosis	Age	Sex	PMI	Braak	Thal	CERAD	NIA-Reagan	αSyn
AD	61	F	48	5–6	5	C	High prob	None
AD	64	M	12	5–6	5	C	High prob	None
AD	85	F	21	5–6	3	B	High prob	None
AD	90	F	11	5	3	B		None
AD	63	F	48	6	5	C		None
C	88	M	39	0	0	0		None
C	88	F	22	2	0	0		In S Nigra
C	63	M	30	0	0	0		None
C	90	M	30	0	1	0		None
C	91	M	27	3	4	C		None

5 severe Alzheimer’s disease cases (Braak stages V–VI) and 5 nondemented cases (Braak stages 0–III) were used to determine the presence of oAβ in brain derived exosomes

### Immunohistochemistry and immunofluorescence of brain sections

Formalin-fixed, paraffin-embedded 10 μm sections of temporal neocortex from healthy control and AD brains (Table 1) were used for this study and pretreated as previously described [6]. After 5 min blocking of endogenous peroxidase by incubation in Background Sniper (Biocare Medical), slides were washed in Tris-buffered saline (TBS) solution and incubated with rabbit polyclonal anti-flotillin-1 (Abcam, 1:200) followed by incubation with either the second primary mouse monoclonal antibody (mAb158, 1:7500, BioArctic) or mouse monoclonal antibody 82E1 (1:25, IBL International) for 30 min at room temperature (RT).

After rinsing in TBS, the slides were incubated for 30 min with MACH 2 Double Stain (Biocare Medical). Following this step, a 30 min incubation was performed with an AP linked chromogen IP Warp red/HRP linked chromogen Vina Green cocktail (Biocare Medical). After rinsing in deionized water, the sections were counterstained with Mayers haematoxylin (Histolab Products AB) and mounted with Pertex mounting media (Histolab Products AB) and micrographs were obtained using 100 × oil immersion objective (Nikon Eclipse 80i, Digital Sight DS-Fi1).

After blocking and incubating with primary antibodies as described above, the slides were rinsed in TBS and incubated with a Fluorescence Enhancement Probe Mouse/Fluorescence Enhancement Probe Rabbit cocktail (Biocare Medical) for 20 min subsequent to a 40 min incubation with a Goat-anti-Mouse DyLight 549/Goat Anti-Rabbit DyLight 488 cocktail (Biocare Medical) diluted 1:200, respectively. After rinsing in deionized water, the sections were nuclear counterstained with DAPI and mounted with Fluoro Care Anti-Fade Mountant (Biocare Medical) and analysed with a Zeiss LSM 700 confocal microscope. The differential interface contrast (DIC) mode and 405, 488, 555 and 639 lasers were used to acquire the images with 63x/1.40 oil immersion plan-apochromatic DICII objectives. The micrographs were processed using Huygens (Scientific Volume Imaging) and ZEN lite (blue edition) software.

### Cell lines and differentiation

Two different cultured cell types were used in the study: the human-induced pluripotent stem cells, AF22 (from a control subject) and the human neuroblastoma cell line, SH-SY5Y (ECACC: Sigma-Aldrich). The neuroepithelial stem cell line, AF22, derived from human-induced pluripotent stem cells from human skin fibroblasts was provided by Dr. Anna Falk, Karolinska Institute, Sweden. The process of reprogramming human cells was approved by the Ethical Committee at Karolinska Institute, Sweden (dnr 2012/208-31/3 with addendum 2012/856-32). All samples were given with informed consent. The AF22 cell line has previously been shown to have a stable neuronal differentiation competence and the capacity to generate functionally mature human neurons [20], denoted hiPSC. The hiPSCs were cultured on 0.01% poly-l-ornithine and laminin (10 µg/mL, Sigma-Aldrich) coated cell culture flasks (Corning) in DMEM/F12 media (Gibco by Life Technologies), supplemented with EGF (10 ng/mL, PeproTech), FGF2 (10 ng/mL, PeproTech), N2 (5 µl/ml, Life Technologies) and B27 (1 ml/L, Life Technologies) and further differentiated for 40 days to functionally mature human neurons in 1:1 DMEM, neurobasal media containing B27 (10 µl/ml), Laminin (1 µl/ml) and N2 (5 µl/ml) as previously described [20].

Neuronal differentiation of the human neuroblastoma cell line SH-SY5Y was performed as previously described [1]. In brief, SH-SY5Y cells were cultured and pre-differentiated for 7 days using 10 μM retinoic acid (RA; Sigma-Aldrich; denoted as raSH-SY5Y). Pre-differentiated raSH-SY5Y cells were seeded on 6-, 12- or 24-well glass plates coated with 20% extracellular matrix (ECM) gel (BD Bioscience) and further differentiated for 10 days with serum-free MEM (Gibco by Life Technologies) supplemented with brain-derived neurotrophic factor (BDNF, 50 ng/ml, PeproTech), neuregulin β1 (NRGβ1,10 ng/ml, R&D Systems), nerve growth factor (NGF, 10 ng/ml, R&D Systems) and vitamin D3 (VitD3, 24 nM, Sigma-Aldrich). These fully differentiated cells are denoted dSH-SY5Y.

In addition, SH-SY5Y cells expressing a CD63-EGFP fusion protein were generated using AddGene plasmid #62964.

### Labelling and oligomerization of Aβ1-42

Recombinant Aβ1-42 peptides (Innovagen,) was dissolved in 1,1,1,3,3,3-hexafluoro-2-propanol (HFIP, Sigma-Aldrich) and vacuum dried overnight. Aβ1-42 (1.054 mM final concentration) was resuspended in Na_2_CO_3_ (0.1 M pH 8.5) and incubated with the fluorophore Alexa Fluor 700 (AF700) succinimidyl ester (1.58 mM final concentration, Life Technologies) for 40 min at 4 °C or the fluorophore 6-carboxytetramethylrhodamine succinimidyl ester (TMR, Invitrogen), in a molar ratio of 2:, incubated overnight at 4 °C. Labelled Aβ1-42 was diluted to a final concentration of 100 µM in HEPES 20 mM pH 7.4, vortexed, sonicated for 10 min and incubated at 4 °C overnight. After the overnight incubation, Aβ1-42 was separated from free dye with size exclusion chromatography (SEC). A Sephadex 75 10/300 GL column coupled to a liquid chromatography system (ÄKTA pure, GE Healthcare) was equilibrated with NH_4_HCO_3_ 50 mM pH 8.5 and 500 µl of sample was injected into the column. To estimate the molecular weight of the Aβ species, LMW gel filtration calibration kits (GE Healthcare) were used. Oligomeric and monomeric Aβ species were eluted at a flow rate of 0.5 ml/min, collected and lyophilized. Then, Aβ species (oAβ-AF700) were resuspended in phosphate-buffered saline (PBS) solution and quantified spectrophotometrically at 215 nm by using the Aβ1-42 extinction coefficient (Aβ1-42 ɛ214 nm = 75,887 M^−1^ cm^−1^) according to Lambert–Beer’s law. Protein aliquots were stored at − 80 °C.

### Exosome purification, characterization and labelling

Isolation of brain exosomes from extracellular space of freshly frozen human brain tissues (250 mg) was performed as previously described [42]. Tissue was dissociated with papain (20 units/ml, 15 min at 37 °C, Sigma-Aldrich) followed by filtration through 40 µm mesh filter (BD Biosciences) and a 0.2 µm syringe filter (Thermo Scientific) to separate extracellular matrix from cells. The crude exosomes were then isolated by differential centrifugation method and subsequently purified by sucrose density gradient as previously described and resuspended in PBS, lysis buffer or diluent C (Sigma-Aldrich) for further experiments [42].

Exosomes from conditioned media of raSH-SY5Y cells were isolated by differential ultracentrifugation. In brief, 50–80 million raSH-SY5Y cells were incubated with oAβ-AF700for 3 h at 37 °C. After PBS washing, cells were kept for 48 h in MEM supplemented with exosome-free serum (System Biosciences). Culture supernatants were collected and spun at 1000 × g for 10 min for removal of cellular debris. The supernatants were then filtered through 0.22 µM filter and sequentially centrifuged at 5000×*g*, 10,000×*g*, and 100,000×*g*. The final pellet was then resuspended in PBS, lysis buffer or diluent C for further analysis.

Exosomes were labelled with PKH67 or PKH26 dye (Sigma-Aldrich), according to the manufacturer’s protocol. Briefly, 4 μL PKH67 dye was mixed with exosome suspension in diluent C and incubated for 10 min at 37 °C. The labelling reaction was stopped by adding 20 ml chilled PBS. Labelled exosomes were ultra-centrifuged at 100,000×*g* for 70 min, washed with PBS, ultra-centrifuged again at 100,000×*g* and the pellet was resuspended in PBS.

### Cellular uptake of exosomes

dSH-SY5Y or hiPSCs cells were plated on coverslips in the respective serum-free growth medium. Before the uptake assay, exosomes were isolated from brain tissue or conditioned media of raSH-SY5Y cells and labelled with PKH67 or PKH26 as described above. In order to use equal amounts of exosomes in the cell cultures, exosomal protein content was quantitated by using BCA (Bio-Rad) or QuantIT (Invitrogen). Brain exosome abundance was quantified according to the AChE activity (EXOCET Exosome Quantification kit; System Biosciences) according to the manufacturer’s protocol. The uptake was performed by incubating cell cultures with 100 µl of exosome solutions (corresponding to an exosomal protein content of 0.62 ± 0.28 μg/μl from brain or 0.71 ± 0.33 μg/μl from conditioned media; equal to 1.4e10 exosome abundance from brain) in a humid chamber for 3 h (37 °C, 5% CO_2_). For inhibition experiments, cultured cells were pre-incubated for 30 min with the endocytosis inhibitors, dynasore (dynamin inhibitor, 80 µM), phenylarsine oxide (clathrin inhibitor 20 µM), genistein (caveolae inhibitor, 200 µM) all from Sigma-Aldrich. Isolated exosomes in PBS were added to cells for 3 h as above and flow cytometry was performed.

### Co-culture model

Co-culture of donor-recipient cells was performed by using two different methods namely the coverslip system (where physical contact of synapses is possible) or the transwell system (where physical contact of synapses is not possible). In both cases, donor cells (raSH-SY5Y 12,500 cells/cm^2^, or hiPSCs 25,000 cells/cm^2^) were seeded on glass coverslips coated with 0.1 mg/ml poly-l-ornithine and 10 µg/ml laminin and cultured as described above for 3 h at 37 °C with either 1 µM of oAβ-AF700 or labelled exosomes from brain tissue or conditioned media, and thereafter washed twice with PBS.

In the transwell system, donor cells were seeded on a polycarbonate membrane filter with a 0.4 µm pore size (Falcon, Corning), placed on top of recipient cells (dSH-SY5Y) and subsequently co-cultured for 24 h. At the end of incubation, the membrane filter was removed and recipient cells were washed with PBS and analysed with flow cytometry or fixed with 4% PFA for immunofluorescent labelling.

In the coverslip system, the donor cells were seeded on glass coverslips (VWR International) and placed upside down on top of recipient cells, predifferentiated as described above (resulting in donor cells facing recipient cells) and subsequently co-cultured for 24 or 48 h at 37 °C. For gel cultured cells this results in a 3D environment. Thereafter, the coverslips with donor cells were removed and recipient cells were washed with PBS and either analysed with flow cytometry or fixed with 4% PFA for immunofluorescent staining. Additionally, to control for donor cell contamination in the recipient cell samples, donor cells were transfected with BacMam 2.0 early endosomes Rab5a-RFP (Life Technologies) at a final concentration of 30 particles per cell before co-culture and RFP fluorescence was monitored in recipient cells by flow cytometry.

### Immunocytochemistry

Co-localization of oAβ and flotillin-1 and TSG101 were visualized with immunostaining using 1: 5000 solution of mouse anti-mAb158, 1: 200 solution of mouse anti-flotillin-1 and a 1: 200 solution of mouse anti-TSG101. The secondary antibodies were Cy3 conjugated (Jackson Immuno Research, 1:1000) and Alexa Fluor 488-conjugated (Invitrogen, 1:400) goat anti-mouse IgG. GFP was detected using 1:200 rabbit anti-GFP (Life Technologies) and 1:400 goat anti rabbit Alexa flour 647 (Life Technologies).

### Cell microscopy

Images of fixed cells were acquired with a Zeiss LSM 700 confocal microscope. The differential interface contrast (DIC) mode and 405, 488, 555 and 639 lasers were used to acquire the images with 63x/1.40 oil immersion plan-apochromatic DICII objectives. Live cell imaging were done using a Zeiss Primo Vert microscope. The micrographs were processed using Huygens (Scientific Volume Imaging) and ZEN lite (blue edition) software.

### Flow cytometry

To detect PKH67 labelled exosomes or oAβ-AF700, cells were released from the ECM gel using Corning Recovery Solution (Corning) according to manufacturer’s instructions, filtered through CellTrics 30 µm filters (Sysmex), re-suspended in PBS, and subsequently analysed on a BD FACSAria™ (BD Biosciences) flow cytometer.

After inhibiting the gene expression of exosome markers TSG101 and VPS4A in raSH-SY5Y cells, using RNA interference, the number of secreted exosomes were analysed using the Exo-Flow™ kit (System Biosciences, USA), targeting CD9, CD63 and CD81, as per manufacture’s instruction.

### Enzyme-linked immunosorbent assay (ELISA) analysis

Altogether, 96-well EIA/RIA plates (Corning Inc.) were coated at 4 °C overnight with 200 ng/well of mAb158 in PBS. Plates were blocked with 1% bovine serum albumin (BSA) in TBS. Exosome samples prepared in RIPA buffer (150 mM sodium chloride, 1% Triton X-100, 0.5% sodium deoxycholate, 0.1% sodium dodecyl sulfate and 50 mM Tris, pH 8.0) were added to the plates in duplicates and incubated for 2 h at 37 °C. A total of 1 mg/mL of biotinylated mAb158 was added and incubated for 1 h at 22 °C, followed by 1 h at RT incubation of streptavidin-coupled poly-HRP (Mabtech). K-blue enhanced (ANL product, Sweden) was used as HRP substrate and plates were read in a spectrophotometer at 450 nm, using Spectra MAX 190 and then analysed with SOFT Max Pro. Wells were washed three times in TBST between each step. The 82E1 sandwich ELISA was performed same as above using 0.25 µg/ml for capture and detection antibody [56]. The amount of oAβ within exosomes was quantified with respect to a standard curve created with serial dilution of synthetic Aβ oligomers and expressed as picomolar/mg of protein.

### Immunoblot analysis

Exosomes were prepared as described above. Brain lysates were prepared from homogenised brain tissue followed by addition of lysis buffer (150 mM NaCl, 0.5% deoxycholate, 1% Triton X-100, 50 mM tris-HCL pH 7.5, 20 μl/ml phosSTOP (Roche), 10 μl/ml Halt Protease inhibitor cocktail (Thermo Fisher Scientific)), clarified by centrifugation at 10,000 x g for 5 min and sonicated using an ultrasonic probe. Cell lysates were prepared from cells collected in lysis buffer followed by homogenisation and sonication. Samples were mixed with 4x Laemlli loading buffer and separated on a ClearPAGE SDS Gel 4–12% or 10% (C.B.S. Scientific), and transferred onto a nitrocellulose membrane (Invitrogen). The gel was subsequently stained using InstantBlue protein stain (Expedeon). Additionally, exosomes, isolated from Control and AD brains or conditioned media of oAβ-AF700-treated raH-SY5Y cells were lysed by freeze-thawing and subsequently run by SEC using the conditions described above. The eluted proteins were collected in fractions of 1 ml, lyophilized, resuspended in 15 µl PBS and spotted on 0.2 µm nitrocellulose membrane. Membranes were then blocked by 3% BSA followed by primary antibody incubation. The following antibodies were used: anti-flotillin-1 (1: 500, BD Transduction Laboratories); anti-alix (1: 1000, EMD Millipore); anti-TSG101 (1:1000, Thermo Fisher Scientific); 1:1000, anti-VPS4A (Abcam), anti-calnexin (1:1000, Abcam), anti-synaptophysin (1:1000, Synaptic Systems), mAb158 (1: 5000/10000, BioArctic) and anti-glyceraldehyde 3-phosphate dehydrogenase (GAPDH, 1:40000, Synaptic Systems,). Anti-rabbit IgG, horseradish peroxidase (HRP)-linked antibody (1:3000, Dako) and anti-mouse IgG, HRP-linked antibody (1:3000, Dako) were used as secondary antibodies. The blots were visualized using Amersham™ ECL™ (GE Health Care) or SuperSignal^®^ (Thermo Scientific) detection systems and analysed by ImageJ software.

### Negative staining and transmission electron microscopy of exosomes

Exosome suspensions were fixed in 4% paraformaldehyde (at 1:1 dilution, for a final paraformaldehyde concentration of 2%) overnight at 4 °C and stored at − 20 °C until use. Thawed exosome suspensions were vortexed briefly and centrifuged in a microcentrifuge for 30 s. Exosomes were adsorbed on Formvar-coated Ni mesh grids by placing the grids on 5 µl drops of exosome suspension for 20 min in a dry chamber. Negative staining was performed by gently dripping 100 µl 2% aqueous uranyl acetate onto the grid, followed by removal of excess uranyl acetate solution using a lens paper. The grids were examined in a JEOL JEM-1230 electron microscope at 100 kV accelerating voltage. Electron micrographs were obtained at 150,000–200,000 × magnification, for a final image scale of 3.1–4.2 pixels/nm.

### Tunable resistive pulse sensing by qNano

Exosome size and particle number were analysed by TRPS analysis using a qNano instrument (IZON Science, UK) as described previously [37]. First, isolated exosomes from brain tissue or conditioned media of dSH-SY5Y cells were diluted and passed through a 0.2 μm filter (Millipore). Subsequently, particle numbers were counted for a maximum of 5 min or until 500 particles had been counted, using 8 mbar pressure and the NP150 or NP100 nanopore membranes with a stretch between 45 and 47 mm. Voltage was set to 0.1 and 0.25 mV to achieve a stable current. Particle size histograms were recorded when root mean square noise was below 13 pA and particle rate in time was linear. Calibration was performed using known concentration of beads CPC70D (mode diameter 70 nm) or CPC100B (mode diameter: 110 nm) (all from IZON) diluted in 1:500 0.2 μm filtered PBS.

### Proteinase K digestion

To examine whether exosome-associated Aβ is luminal or bound to the exterior exosome surface, exosomes were isolated from conditioned media (oAβAF700-treated) of dSH-SY5Y cells and incubated with proteinase K (Sigma-Aldrich,1 mg/ml) for 30 min at 37 °C. 4-(2-aminoethyl)-benzene-sulfonyl fluoride (Sigma-Aldrich, 0.5 mM) was subsequently added to the vesicle fraction to inactivate the enzyme prior to two rounds of 100,000 × g centrifugation. The final pellet was resuspended in PBS and AF700 fluorescence was measured in Tecan Safire2 microplate reader at Ex/Em 696/719 nm.

### Cytotoxicity assay

To investigate the toxic effect of exosomes on neurons, equal amounts of exosomes (based on exosomal protein estimation by BCA) from brains or cells were added to dSH-SY5Y cells and hiPSCs in our co-culture model for 48 h, as described above. At the end of incubation, donor cells were removed and cell medium was collected to assess the release of lactate dehydrogenase (LDH) in the medium. Collected medium was centrifuged 2000×*g* for 5 min at 4 °C and LDH assay (Pierce) was performed according to manufacturer’s instructions. The absorbance was measured in a microplate reader (SpectraMAX 190) at 490 nm with subsequent blank at 680 nm. Furthermore, XTT (2,3-bis [2-methoxy-4-nitro-5-sulfophenyl]-5-[(phenylamino) carbonyl]-2H-tetrazolium hydroxide) assay using the Cell Proliferation Kit II (Roche Diagnostics GmbH) was performed on acceptor cells according to the manufacturer’s instructions. The reduced XTT product produced by mitochondrial enzymes in viable cells, formazan (bright orange in colour) was measured after 8 h of incubation at 450 and 750 nm using a Victor 3 V 1420 multilabel plate reader (PerkinElmer). Both LDH and XTT values were presented as percentage of untreated control.

### RNA interference

Cells were seeded at a density of 12 500 cells/cm^2^ in a 6-well plate and transfected 24 h later with TSG101 or VPS4 mRNA-targeting siRNA or a non-targeting siRNA with no homology to any known human gene (All Stars Negative Control siRNA) with the HiPerFect transfection reagent (all from Qiagen) according to manufacturer’s protocol. TSG#6 (CAGTTTATCATTCAAGTGTAA), TSG#3 (ACTGTCAATGTTATTACTCTA), VPS#7 (AAGCTGAAGGATTATTTACGA) and VPS#5 (CTCAAAGACCGAGTGACATAA) siRNAs were used for this study. The final siRNA concentrations in the culture medium ranged from 10 to 20 nmol/l. Twenty-four hours after transfection, knockdown was verified by quantitative real-time PCR and Western Blot analyses, and a decrease in the mRNA level of 70% or greater was considered sufficient downregulation.

### Gene expression analysis

Total RNA was extracted with the RNeasy Mini Kit (Qiagen), and cDNA was obtained with the High Capacity RNA-to-cDNA Kit (Applied Biosystems). The expression levels of TSG101 or VPS4 mRNA were analysed with a 7500 Fast Real-Time PCR system and FAM/MGB probes (Applied Biosystems) to confirm downregulation after siRNA treatment. All reactions were performed according to the manufacturer’s instructions. GAPDH was amplified as an internal standard. The data were calculated according to the comparative Ct method to present the data as fold differences in the expression levels relative to the control sample.

### Statistics

All statistical analyses were performed using GraphPad Prism Software. Data were expressed as the mean ± SEM, and statistical comparisons were made using two-tailed unpaired Student’s *t* tests with Welch’s correction or one-way ANOVA with Tukey's correction. Every batch of cell cultures was treated as one independent experiment (*n* = 1). *P* values less than or equal to 0.05 were deemed statistically significant.

### Study approval

The collection and use of post mortem brain tissue was approved by the Regional Ethical Committee in Uppsala, Sweden (2005/103, 2005-06-29; 2009/089; 2009-04-22). The process of reprogramming human cells was approved by the Ethical Committee at Karolinska Institute, Sweden (dnr 2012/208-31/3 with addendum 2012/856-32). A written informed consent was received from all donors.

## Results

### Alzheimer brain exosomes are enriched with oAβ

**Fig. 1:**
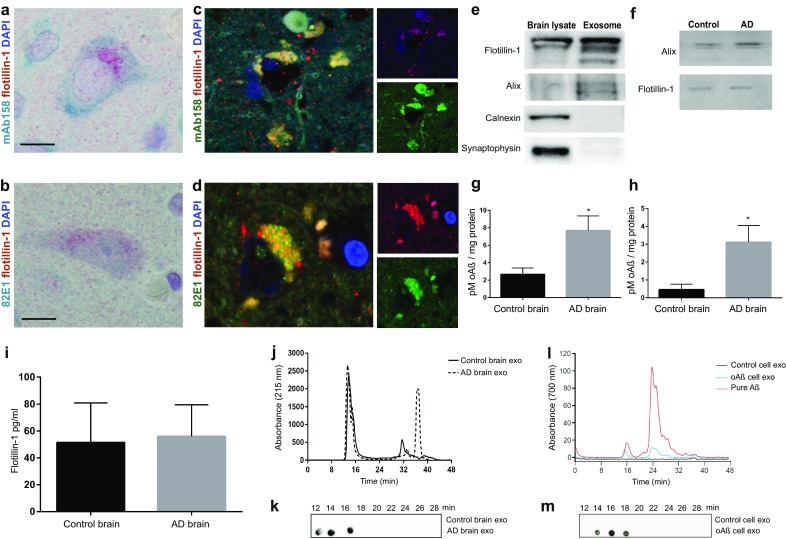
AD brain exosomes are enriched with oAβ. AD brain sections from temporal neocortex show co-localization of probable oAβ to exosomes. **a**, **b** Double-immunostaining with the exosome marker flotillin-1 (labelled red), and two different oligomer-selective (see main text) Aβ antibodies mAb158 and 82E1, respectively (labelled blue-green). Scale bar 10 µm. **c**, **d** Co-localization of oAβ to exosomes was performed after immunofluorescence labelling. mAb158 or 82E1, respectively, showed substantial co-localization (yellow) with flotillin-1 inside cells in the AD brain (*R*^2^ = 0.78 and 0.86 for mAb158 and 82E1, respectively). Scale bar, 5 µm. **e** Immunoblot showing flotillin-1, alix, calnexin and synaptophysin in exosome and brain lysate, demonstrating no cellular or synapse vesicle contamination in the exosome preparation. Loading control is shown in Supplementary Fig. S1d. **f** Immunoblot demonstrating the presence of flotillin-1 and alix, in exosome fractions isolated from control and AD brain. Quantitative ELISA analysis of oAβ in exosomes isolated from AD and control brains, using 82E1 (**h**) and mAb158 (**g**) antibodies, respectively. Data are presented as the mean picomolar of oAβ per mg total exosome protein ± SEM (*n* = 3). *p < 0.05 by two-tailed unpaired Student’s *t* tests with Welch’s correction. **i** Quantitative analysis of flotillin-1 by ELISA in control and AD brain exosomes showing equal amount of flotillin-1 between the groups. **j** Representative SEC profile of lysed exosomes isolated from control and AD brain samples at 215 nm absorbance (general protein detection). **k** Detection of oAβ in SEC eluate fractions of AD and control brain exosomes by dot blot using mAb158 antibody. **l** SEC chromatograms of exosomes isolated from conditioned media of control- or oAβ-AF700 treated dSH-SY5Y cells as well as pure oAβ-AF700. Detection at 700 nm absorbance (AF700 detection). **m** Dot blot detection of oAβ (mAb158 antibody) in SEC eluate fractions of exosomes isolated from conditioned media of control- or oAβ-AF700 treated dSH-SY5Y cells of control and oAβ-AF700 treated dSH-SY5Y cells exosomes. The analysis of SEC fractions confirms the presence of oAβ in exosomes

To examine whether oAβ has the potential to localize to cellular structures that form exosomes in the AD brain we analysed the co-localization of potential oAβ labelling and the marker flotillin-1, expressed in multi-vesicular bodies and exosomes. Brain sections from temporal neocortex from four post mortem AD brains were double-immunostained with flotillin-1 and one of two different Aβ antibodies (mAb158 or 82E1). Cells with neuronal morphology displayed both mAb158 or 82E1 and flotillin-1 labelling (Fig. 1a, b). Next, the possible co-localization of mAb158 or 82E1 with flotillin-1 was analysed on immunofluorescently labelled, fixed brain sections using the same antibodies. Although the resolution is not sufficient to prove co-localization, we observed a strong correlation between mAb158 or 82E1 and flotillin-1 labelling, suggesting a subcellular co-localization (Fig. 1c, d) (*R*^2^ = 0.78 and 0.86 for mAb158 and 82E1, respectively). The detected fluorescence was not due to lipofuscin-derived autofluorescence (Supplementary Fig. S1a). Currently, no antibodies with verified oAβ selectivity in IHC applications have been reported. Thus, we selected the two Aβ antibodies with most extensive data on oligomer selectivity in other assays (mainly ELISA). Both these antibodies showed similar labelling patterns in brain tissue (Fig. 1a–d). Furthermore, our findings from dot-blot (Fig. 1k, m, described below) supports that mAb158 has a selectivity for aggregates over monomers also in assays where the protein is in a bound state. Taken together, these data indicate that the identified Aβ labelling likely represents oligomeric Aβ aggregates. Thus, our findings suggest an association of oAβ with intracellular compartments containing flotillin-1, including multivesicular bodies, in the human AD brain, which thus suggests that oAß could be released in exosomes.

Since oAβ is believed to be central to AD pathophysiology [50], we postulated that exosomes from AD brains would contain higher concentrations of oAβ when compared to similar preparations of control brain samples from patients deceased from non-neurological reasons. Using well-established methods for mild dissociation of nervous tissue followed by separation of extracellular matrix from cells we were able to isolate exosomes by sequential ultracentrifugation from fresh-frozen, post mortem brain tissues from temporal neocortex of both AD and control subjects. Analyses of the isolated fractions by Tunable Resistive Pulse Sensing technology (TRPS, qNano system) and transmission electron microscopy (TEM, Supplementary Fig. S1b, c) demonstrated presence of vesicles with a size corresponding to exosomes [55]. Immunoblotting confirmed the extracellular origin of the extracted vesicles by presence of the exosomal markers alix and flotillin-1 and the lack of the cellular marker calnexin and the synapse vesicle marker synaptophysin (Fig. 1e). Furthermore, immunoblotting confirms the presence of exosomal markers in extracted vesicles from both control and AD brains (Fig. 1f). As immunostaining of flotillin-1 and oAβ in the AD brain sections does not have the sufficient resolution, ELISA, which is a more quantitative method, was implemented to demonstrate the oAβ in brain exosomes. When used as capture and target in an ELISA the Aβ antibodies mAb158 is selective for Aβ oligomers [19, 57], while 82E1 detects both soluble and fibril Aβ [31]. Interestingly, ELISA with either one of these two oligomer-selective antibodies showed significantly higher levels of oAß in exosomes from AD brains (*n* = 5, 3.12 ± 0.93 pM oAß/mg protein for 82E1 and 7.69 ± 1.68 pM oAß/mg protein for mAb158) than in those from healthy control brains (*n* = 5, 0.46 ± 0.30 pM oAß/mg protein for 82E1 and 2.66 ± 0.74 pM oAß/mg protein for mAb158) (Fig. 1g, h). There was no significant difference in the amount of flotillin-1 in exosomes derived from AD or control brain tissue of equivalent weight (Fig. 1i). The increase in exosomal oAß was not an effect of co-precipitation of free oAß during the extraction as spiking exosomes with exogenous oAß did not result in increased levels, which also implies that association of oAß with exosomes occurs intracellularly (Supplementary Fig. S1e). Additionally, the presence of oAβ in isolated exosomes was further assessed using size exclusion chromatography (SEC). SEC chromatograms of the isolated and lysed exosomes from control and AD brain samples were analysed at 215 nm absorbance (general protein detection) and proteins were detected in the void volume (first peak) as well as in fractions corresponding to lower molecular weight (Fig. 1j). The presence of oAβ in the different SEC eluate fractions was further investigated by dot-blot analysis using mAb158. This confirmed that exosomes isolated from AD brain samples contained oAβ in SEC fractions running at the oligomer size that was not detected in control brain (Fig. 1k). Similarly, lysed exosomes isolated from conditioned media of control- or oAβ-AF700 treated neuronally differentiated neuroblastoma cells (dSH-SY5Y) as well as pure oAβ-AF700 were analysed on SEC chromatograms with detection at 700 nm absorbance (AF700 detection). This shows absorbance peaks (oligomeric and monomeric) at the same time points (size) for exosomes from conditioned media of oAβ-AF700 treated cells as for the pure oAβ-AF700 initially used to treat the cells (Fig. 1l). oAβ was detected only in fractions from exosomes from conditioned media of oAβ-AF700 treated dSH-SY5Y cells and not in control cell exosomes (Fig. 1m). Furthermore, these findings show that, in addition to previously shown Aβ oligomer selectivity in ELISA assay [19, 57], the mAb158 antibody is oligomer selective also in an assay where protein is not free floating.

The localization of oAß to exosomes was further investigated in neuronal cell models. dSH-SY5Y or hiPSC were incubated with oAβ-AF700 for 3 h, after 48 h exosomes were isolated from the conditioned media by sequential ultracentrifugation. Similar to the brain extractions, the isolated vesicles were characterized as exosomes using immunoblotting for the exosome markers, alix and flotillin-1, lack of cellular marker calnexin and synapse vesicle marker synaptophysin, TRPS and TEM (Supplementary Fig. S2a–d) [35, 54]. To investigate the location of oAß, exosomes isolated from conditioned media from oAß-AF700 treated retinoic acid differentiated SH-SY5Y cells (raSH-SY5Y) were treated with proteinase K (1 mg/ml) to digest proteins on the surface, confirming that the localization of oAβ is mainly luminal as this did not significantly decrease exosomal oAβAF700 (Supplementary Fig. S2e).

### Exosomes from AD brain are internalized and transferred by neurons, causing cytotoxicity

**Fig. 2:**
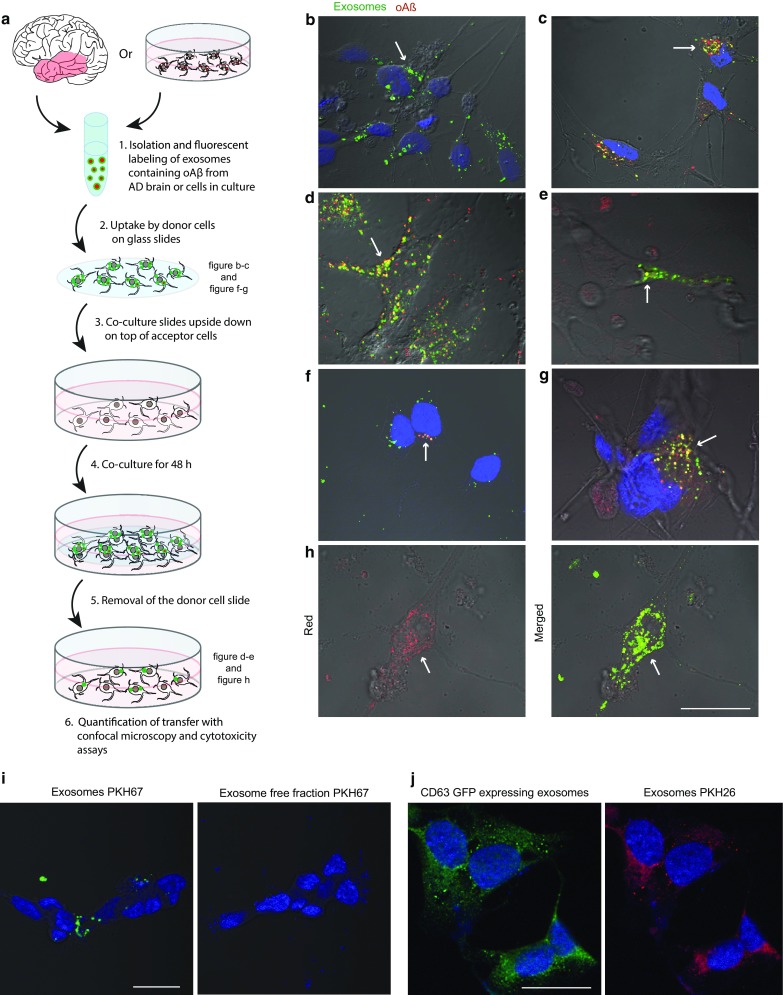
Exosome-mediated uptake and propagation of oAβ in neuronal cells. Exosomes isolated from brain tissue or conditioned media of dSH-SY5Y cells were labelled with the dye PKH67 and added to donor hiPSCs or dSH-SY5Y cells. After 3 h of incubation at 37 °C, donor cells were fixed, stained with mAb158 (for brain exosomes) and analysed by confocal microscopy or donor cells were co-cultured with another set of hiPSCs or dSH-SY5Y (recipient cells). After 48 h of co-culture, donor cells were removed and recipient cells were fixed, stained with mAb158 (for brain exosomes) and analysed by confocal microscopy. **a** A cartoon illustrating the co-culture model with hiPSC or dSH-SY5Y cells employed to measure the transfer of the brain or cell exosomes containing oAβ. Uptake of **b** control and **c** AD brain exosomes (green) containing oAβ (red) in hiPSC donor cells. Transfer of AD brain exosomes (green) containing oAβ (red) to recipient **d** hiPSCs and **e** dSH-SY5Y cells. Uptake of exosomes (green) containing oAβ-AF700 (red) in donor **f** dSH-SY5Y and **g** hiPSCs. **h** Transfer of oAβ-AF700 containing exosomes in recipient dSH-SY5Y cells. Super-imposed image of the red (oAβ) and green (exosomes) channels on a DIC image shows co-localization (yellow) of exosomes and oAβ. Arrows indicate exosomes or exosome containing oAβ. **i** Cellular uptake of isolated brain exosomes and brain exosome free fraction after PKH67 staining showing no PKH67 uptake in the absence of exosomes. **j** Isolated exosomes from CD63-GFP expressing SH-SY5Y cells, double-labelled with PKH26 (red), were added to dSH-SY5Y cells. The CD63-GFP were intensified using an anti-GFP antibody. Scale bar (**b**–**h)**, **j** 20 µm, **i** 10 µm

To investigate whether exosomes could be a vehicle for the spreading of oAß, we investigated if exosomes isolated from AD brains could be taken up by hiPSCs and dSH-SY5Y cells (Fig. 2a). The cells were incubated with exosomes (PKH67 labelled) isolated from brain tissue. After 3 h, both exosomes (Fig. 2b) and oAß (mAb158, Fig. 2c) had been taken up by the cells. Using a co-culture system with hiPSCs or dSH-SY5Y cells, previously shown to result in synaptically connected neurons [40], we next investigated the possible transfer of brain-derived exosomal oAß between these neurons. When RFP labelled donor cells that had been exposed to exosomes were placed on top of recipient neurons for 48 h, a substantial number of transferred exosomes (PKH67) and oAβ (mAb158) could be detected in both types of recipient cells (Fig. 2d, e). Moreover, transferred oAß was still partly co-localized with PKH67 labelled exosomes in the recipient cells, suggesting that after being internalized part of the exosomes can be transferred onwards, still intact with their oAß content. Similarly, uptake of oAß in raSH-SY5Y treated with oAβ-AF700 (3 h) resulted in a co-localization with intracellular flotillin-1 and TSG101 (Supplementary Fig. S3a-b). Isolated (48 h conditioned media) and labelled (PKH67) exosomes were readily taken up by new cells. A high degree of internalization of exosomes in either dSH-SY5Y (Fig. 2f) or hiPSC (Fig. 2g) cells was observed. These vesicular structures had a mainly perinuclear localization and many of them were positive for oAβ-AF700. Using these secondary neurons as donor cells and co-culturing them with a new set of dSH-SY5Y recipient cells for 24 h showed further spread of AF700 labelled oAβ to this third set of cells (red channel, Fig. 2h). Similarly to what we observed for exosomes from AD brains, we could detect a substantial fraction of oAβ that was still co-localized with exosomes (merged, Fig. 2h). These findings support that, apart from releasing their cargo, intact exosomes also can carry and transfer oAβ further to new recipient cells. Likewise, exosomes isolated from conditioned media from cells that had not been exposed to oAβ were also taken up by cells and transferred onwards (Supplementary Fig. S3c), corroborating that the release and uptake of exosomes per se is not dependent on the presence of oAß. To confirm that the observed PKH labelling is not due to artefacts we used the supernatant from the last ultracentrifugation wash-step of the exosome isolation from brain or conditioned media, respectively. The supernatant was labelled with PKH67 using the same protocol as for labelling the exosomes. The labelled sample were subsequently added to raSH-SY5Y cells. Contrary to the exosome fraction the exosome-free fraction did not result in any labelling in the cells either in brain (Fig. 2i) or conditioned media extracts (Supplementary Fig. S3d). Furthermore, isolated exosomes from CD63-GFP expressing raSH-SY5Y cells were double-labelled with PKH26 (red) and added to raSH-SY5Y cells (Fig. 2j). This confirmed that the exosomes studied here are not artefacts, but indeed exosomes.

Further evidence for the exosomal transportation of oAβ was obtained by using a transwell co-culture model after uptake of oAβ containing exosomes to donor cells. Also, in this model oAβ transferred to recipient cells, suggesting vesicle transfer of oAβ between cells without direct neuritic connections. However, compared to the coverslip co-culture model significantly less neurons containing transfer of oAβ was detected in the transwell model, a decrease from 23.60 to 5.18% as analysed by flow cytometry and illustrated by confocal imaging (Supplementary Fig. S4a, b). Thus, suggesting that transfer is more efficient between cells in proximity, which could potentially depend on multiple mechanisms of transfer.

**Fig. 3:**
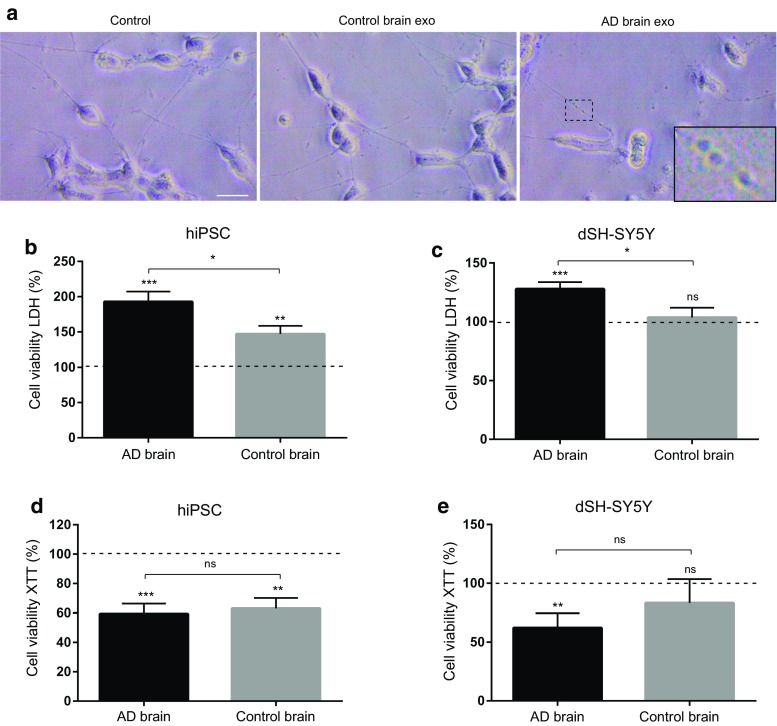
Transfer of AD brain exosomes causes cytotoxicity. Exosomes isolated from control and AD brain tissues were added to donor hiPSCs or dSH-SY5Y cells. After 3 h of incubation at 37 °C, donor cells were washed with PBS and co-cultured with another set of hiPSCs or dSH-SY5Y (recipient cells). After 48 h of co-culture, donor cells were removed. **a** Morphological changes assessed in recipient dSH-SY5Y showing loss of neurite branching after transfer of AD brain exosomes. Also, neurite beading was seen in dystrophic neurites as shown in magnified insert. The conditioned media was collected for LDH assay (**b**, **c)** and recipient cell viability evaluated by XTT (**d**, **e**). Values are expressed as percentage of untreated control. Values are mean ± SEM (*n* = 6 separate experiments). LDH shows that transfer of AD brain exosomes causes significant higher cytotoxicity compared to control brain exosomes in both cell types. NS, not significant; *p < 0.05, **p < 0.01, ***p < 0.001 by two-tailed unpaired Student’s *t* tests with Welch’s correction

Next, we investigated if the spread of exosomes and their oAß cargo could result in neuronal toxicity. Isolated brain exosomes (equalized by protein amounts) from either control or AD brains were added to dSH-SY5Y cells for 3 h, these cells were used as donor cells in 48 h co-culture with recipient dSH-SY5Y cells. The recipient cells were subsequently investigated for morphological changes compared to control co-culture with untreated donor cells (Fig. 3a). As described previously [2] the control dSH-SY5Y shows neuronal morphology with long, branching networks of neurites. Similar morphology was seen in recipient cells in the control brain exosome conditions. Occasional changes in neurite morphology could be identified. Interestingly, more pronounced pathology was seen in recipient cells co-cultured with AD brain exosome treated donor cells. Here dystrophic neurites with neurite beading and loss of neurite branching were identified, known early signs of neurodegeneration [51]. We quantified these effects further and notably, we found that the transfer of AD brain exosomes induced significant cytotoxicity compared to control brain exosomes, as assessed by the degree of membrane leakage of lactate dehydrogenase enzyme (LDH) from the recipient hiPSC and dSH-SY5Y cells (Fig. 3b, c). Induced cellular toxicity was also detected by XTT (2,3-bis [2-methoxy-4-nitro-5-sulfophenyl]-5-[(phenylamino) carbonyl]-2H-tetrazolium hydroxide) analysis in both cell types (Fig. 3d, e). Similar toxicity was further seen after treatment with exosomes from conditioned oAß media (Fig. 5d). In addition, exosomes from control brains which should contain significantly less oAß, caused toxicity in hiPSCs but not in dSH-SY5Y cells (Fig. 3b, e). No toxicity was seen after treatment with exosomes from conditioned media from cells not treated with oAβ (Supplementary Fig. S5a). Taken together, these results suggest that intact AD brain exosomes, carrying oAβ, are taken up by neurons and migrate to second order neurons where they can release their cargo and cause cytotoxicity.

### Inhibition of exosome formation and secretion inhibit the spread of oAβ

**Fig. 4:**
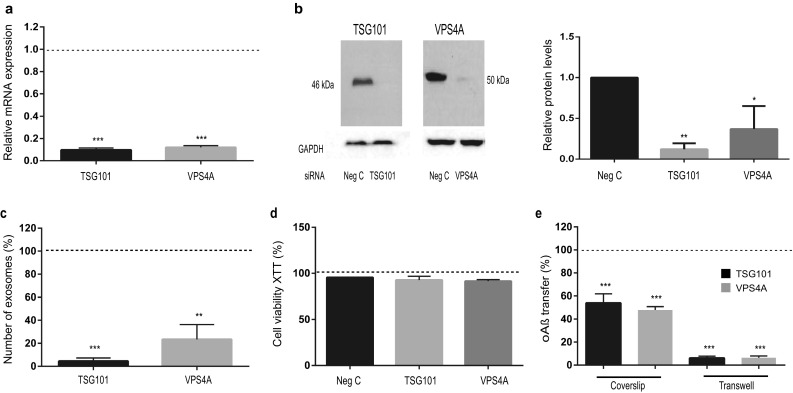
Downregulation of exosomal proteins TSG101 and VPS4A inhibits the spread of oAβ. Depletion of TSG101 and VPS4A by siRNA in dSH-SY5Y cells. **a** Real time PCR analysis of mRNA expressions to show knock down efficiency of transfected cells by TSG101 or VPS4A siRNA. **b** Representative immunoblot picture of cell lysates after siRNA treatment for 72 h and associated densitometric analysis (*n* = 3). **c** Bead flow cytometry analysis of exosomes shows a significant decrease in the number of secreted exosomes after TSG101 or VPS4A siRNA treatment in raSH-SY5Y cells. **d** No cytotoxicity was detected by XTT assay after 48 h of transfection with siRNA. **e** Quantification of oAβ transfer in presence of TSG101 or VPS4A siRNA in both coverslip and transwell co-culture model by flow cytometry, shows that both siRNAs significantly inhibit oAβ transfer. Values are expressed as percentage of siRNA negative control and indicated as dotted line. Values are the mean ± SEM (*n* = 4 separate cultures), *p < 0.05, **p < 0.01, ***p < 0.001 by two-tailed unpaired Student’s *t* tests with Welch’s correction

If exosomes are capable of transferring oAß between neurons, it should be possible to stop this transfer by inhibiting the formation of exosomes. As previously shown, biogenesis of exosomes and its cargo proteins can be modulated by knocking down two Endosomal Sorting Complexes Required for Transport (ESCRT) proteins, TSG101 and VPS4A, required for exosome formation and secretion, respectively [3, 18, 21, 30]. By using siRNA oligonucleotides, a significant decrease in the mRNA levels (decreased in average with 90% for TSG101 and 88% for VPS4A), protein levels (decreased in average with 87% for TSG101 and 65% for VPS4A) and the number of secreted exosomes (decreased in average with 95% for TSG101 and 76% for VPS4A) (Fig. 4a–c) was achieved. The siRNA treatment did not affect viability, as analyses showed that > 95% of the cells were viable as measured by XTT assay (Fig. 4d). To distinguish between different mechanisms of transfer, we again used the coverslip and transwell co-culture models and quantified the oAβ transfer in presence of TSG101 or VPS4A siRNA in our co-culture model using flow-cytometry. Interestingly, upon inhibition of exosome formation and secretion the oAβ transfer was almost completely blocked in the transwell model, whereas almost half of the oAβ transfer could be stopped in the coverslip model (Fig. 4e). These results indicate that exosomes are largely responsible for the neuron-to-neuron transfer of oAβ, although other mechanisms of transfer may also be involved.

### The uptake of exosomes and spread of oAß is dynamin-dependent and can be blocked

**Fig. 5:**
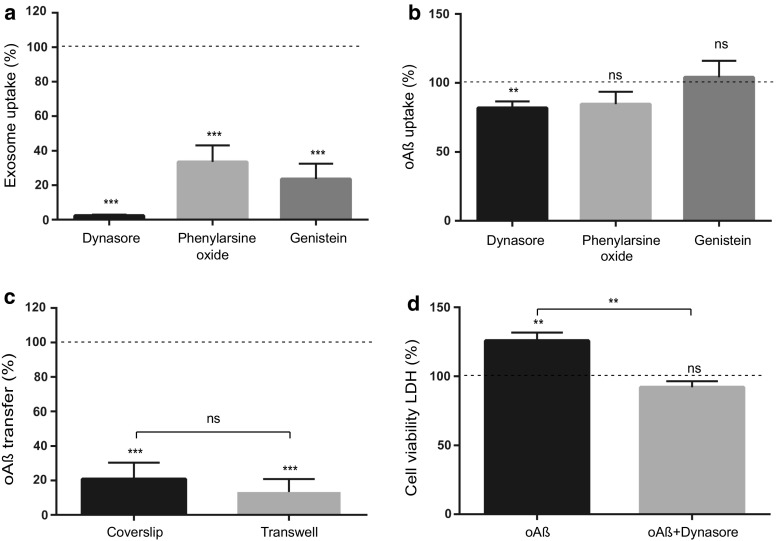
The uptake of exosomes and the subsequent spreading of oAß is dynamin-dependent. **a**, **b** Uptake of PKH67 labelled exosomes or oAβ-AF700 in dSH-SY5Y cells. Cells were pre-incubated with the indicated inhibitors for 30 min, then exposed to exosomes or oAβ-AF700. After 3 h incubation, samples were collected and the proportion of cells with uptake was quantified by flow cytometry and related to untreated control (dotted line). **c** Flow cytometry analysis of oAβ transfer in presence of dynasore in coverslip and transwell co-culture models. After dynasore treatment there is a significant decrease of the proportion of cells with oAβ transfer in both models (control, dotted line). **d** Transfer of exosomes isolated from oAβ treated cells causes cytotoxicity in recipient cells compared to untreated control as shown by LDH assay, whereas dynasore treatment significantly reduces the cytotoxic effect versus untreated control (dotted line). Data are represented as the mean ± SEM, NS, not significant; *n* = 4; **p < 0.01, ***p < 0.001 by two-tailed unpaired Student’s *t* tests with Welch’s correction

After being released, the exosomes can be taken up by the recipient cells via different mechanisms. In dSH-SY5Y cells the uptake of isolated exosomes observed at 37 °C, as described above, was completely abrogated at 4 °C (Supplementary Fig. S5b). Thus, the exosomal uptake is an active process. Neuronal cells are capable of different modes of endocytosis, ranging from receptor-mediated and clathrin-dependent, to independent endocytosis [10, 34, 47]. To explore the uptake mechanism, we treated dSH-SY5Y cells with isolated, PKH67 labelled, exosomes together with various inhibitors of endocytosis: dynasore (dynamin inhibitor), phenylarsine oxide (clathrin inhibitor) and genistein (caveolae inhibitor). Treatment with phenylarsine oxide caused minor cellular toxicity, while no notable cellular toxicity was observed upon treatment with dynasore or genistein (Supplementary Fig. S5c). Strikingly, all three inhibitors caused a significant decrease in the proportion of cells that took up exosomes as quantified by flow-cytometry; the decrease was 97.7% with dynasore, 66.5% with phenylarsine oxide and 76.0% with genistein. Thus, the effect was most marked with dynasore treatment, which almost completely abolished the uptake of exosomes (Fig. 5a), while having only minor effect on the internalization of oAβ added directly to the medium (Fig. 5b). This observation supports that the uptake of exosomes, and thus also oAß transferred via this route, is regulated by dynamin.

The dependence on dynamin, and thus exosomes, for the spread of oAß between neurons was further confirmed using dSH-SY5Y cells in the transwell model using donor cells fed with exosomes, isolated from conditioned media (oAβ-AF700 treated raSH-SY5Y cells). In this setting with an absence of direct neuritic connections, oAβ transfer was almost completely blocked by dynamin inhibition (Fig. 5c), in line with a major dependence on exosomes for transfer. When instead performing the same experiment using the coverslip co-culture model, allowing for direct cell-to-cell contacts, there was also a significant decrease of transferred oAß (Fig. 5c). Since our observations substantiate the hypothesis that exosomes play a significant role in transferring oAβ from one neuron to another, we next sought to investigate whether transfer of exosomes containing oAβ had any direct toxic effect on the recipient cells. Cell-to-cell transfer of exosomes, isolated from conditioned media (oAβ-AF700 treated raSH-SY5Y cells), showed a significant increase in cellular toxicity of 26% in the recipient cell, as evident by LDH assay (Fig. 5d). Exosomes from untreated raSH-SY5Y cells had no significant toxic effect on the recipient cells (Supplementary Fig. S5a). Importantly, we found that such induced toxicity could be abolished by blocking the transfer of exosomes using dynasore (Fig. 5d). These data provide proof-of-concept that the transfer of exosomes and oAβ together with subsequent toxicity, can be prevented by inhibiting the dynamin-dependent uptake pathway.

## Discussion

Recent evidence suggests that toxic Aβ aggregates can spread pathology in the Alzheimer brain. The nature of the propagating species has not been established, although several studies have indicated a particularly pathogenic role of soluble oAβ on synaptic and cellular functions and structure [13, 32, 50, 60]. It has been speculated that exosomes might transfer neurodegenerative proteins in the affected brain [28]. Accordingly, exosomes from blood [22], CSF [17, 49] and cell cultures [46] have been shown to contain monomeric Aβ and tau, but so far, no study has addressed the presence of oAβ in exosomes from human AD brains. In this study, we could show that AD brain exosomes contain an increased amount of oAβ compared to non-neurological control brains and found evidence that exosomes can be responsible for the neuron-to-neuron transfer of toxic oAβ. These findings suggest that exosomes might be the main mediator of the pathogenic progression in AD as was recently suggested for dementia with Lewy bodies [41]. In addition, we found a co-localization between oAβ and exosomes inside neurons, which might indicate that exosomes play a role in Aβ sorting and oligomerization [16]. The AD brain exosomes were further shown to effectively transfer oAβ from one neuron to another, with subsequent toxic effects on the recipient cells. Interestingly, at least a part of the exosomes seems to be transferred intact to further cells, consistent with a recent study showing that a substantial fraction of exosomes internalized in one cell were subsequently passed on to a second cell [44]. Importantly, there is increasing evidence of correlations between intra-neuronal oAβ and cell death [43]. We have previously demonstrated that transfer of oAβ causes neurotoxicity [40] which has also been shown with Aß containing exosomes isolated from AD CSF [33]. Accordingly, we now found signs of neurotoxicity both morphologically and with the LDH and the XTT assays after transfer of exosomes carrying oAβ. This observation not only reinforces the role of intracellular oAβ in AD pathogenesis but also establishes the disease relevance associated with the exosomal neuron-to-neuron transfer of intercellular oAβ.

The molecular content of exosomes is a fingerprint of the releasing cell type and, because of their small size, neuronal exosomes are released into accessible body fluids such as blood and CSF [48]. Since neuronal exosomes display unique neuron-specific surface markers [22, 26] they may be a valuable biomedical marker for early diagnosis and treatment in AD. Indeed, exosomes have recently been highlighted as diagnostic biomarkers in various disease conditions, including AD [29, 36]. In concordance, the finding of increased levels of oAβ in brain exosomes opens the possibility that similar features could be detected also in easily accessible body fluids, such as plasma and CSF. Hence, measurement of increased oAβ in exosomes from such patient samples could potentially serve as a diagnostic tool.

The intercellular propagation of oAβ and its ensuing toxicity could also serve as a potential treatment target by inhibiting either formation, secretion, or cellular uptake of exosomes. Indeed, downregulation of TSG101 and VPS4A, proteins necessary for exosome formation and secretion, was found to result in decreased release of exosomes and a reduced subsequent transfer of oAβ, thus supporting the possibility of modulating this mechanism. Moreover, these observations are in line with recent studies showing that interfering with exosome release can impact the release of specific proteins [9, 14]. An alternative therapeutic target could be the dynamin-dependent uptake of exosomes [23, 24] as the dynamin inhibitor dynasore decreased exosome propagation, spread of oAβ and the associated neuronal toxicity, leading to rescued cell viability. Dynasore itself would not be a feasible therapeutic substance, but phenothiazine-derived antipsychotic drugs have been suggested to inhibit dynamin dependent endocytosis [11] and could thus be suitable for further drug development.

In conclusion, our results point to a role for exosomes in the spreading of toxic oAβ and the associated disease progression in the AD brain (summarized in Supplementary Fig. S6). It has been suggested that exosomal release may provide an alternative disposal mechanism to lysosomal degradation of oAβ [5] or other proteins that are resistant to degradation [55]. We speculate that this alternative mechanism of clearance, which initially could be beneficial for the cells, over time becomes a liability with increased propagation of pathological proteins throughout the brain. The possibility of inhibiting exosome transfer and the related spread and toxicity of oAβ may lead to the identification of new pharmaceutical targets for AD.

## Electronic supplementary material

Below is the link to the electronic supplementary material.

Supplementary material 1 (DOCX 4839 kb)
