# Supplementary material for: Alzheimer’s disease pathology propagation by exosomes containing toxic amyloid-beta oligomers
Source: Acta Neuropathol. 2018 Jun 13;136(1):41–56. doi: 10.1007/s00401-018-1868-1 (PMC6015111; doi:10.1007/s00401-018-1868-1)
Supplement: Supplementary file 1 — Supplementary material 1 (DOCX 4839 kb) [file 401_2018_1868_MOESM1_ESM.docx]

**Supplementary Figure S1.**

**
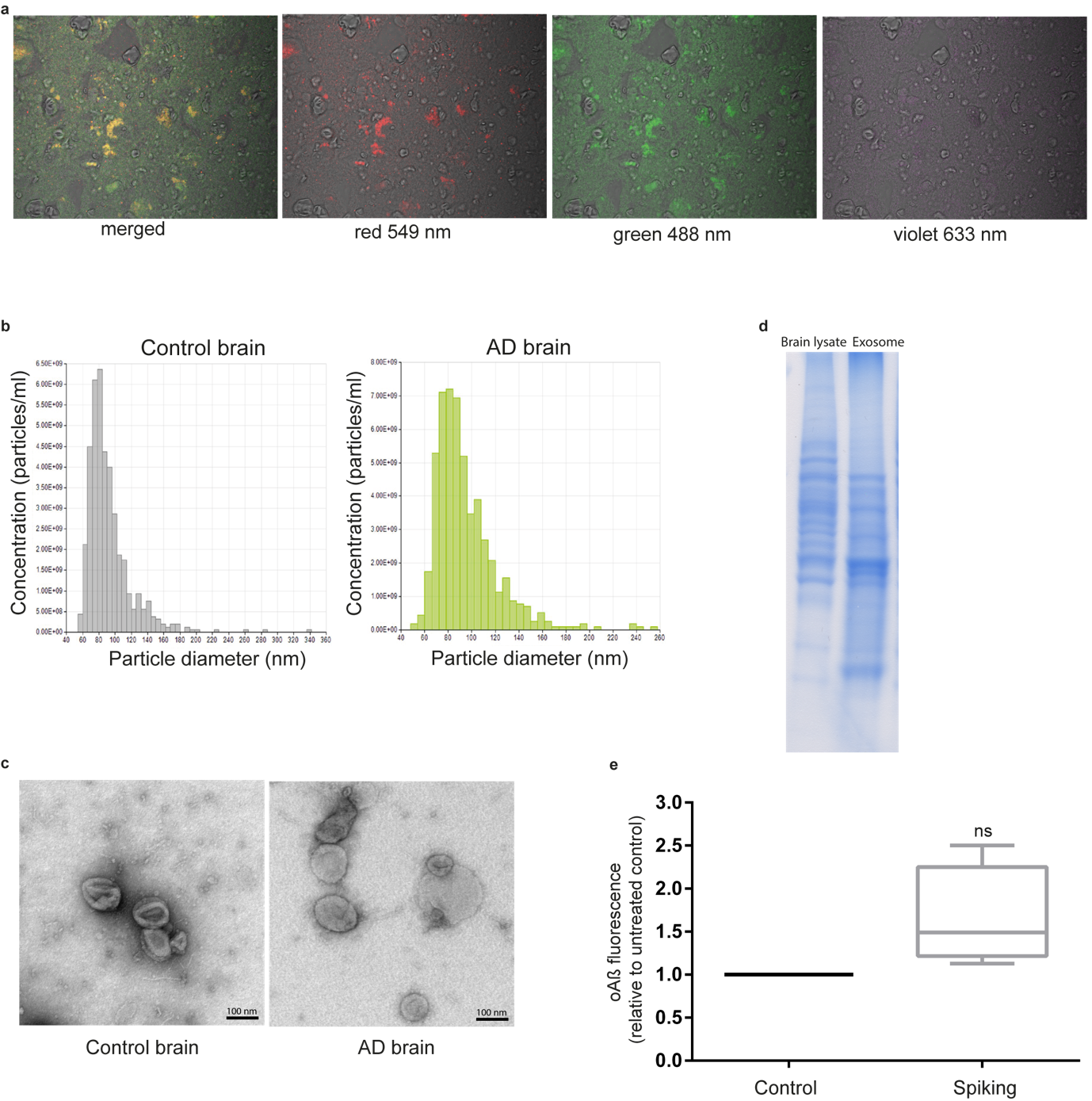
**

**Supplementary Figure S1. Characterization of exosomes isolated from brain tissue
(a)** Far red channel of stained brain tissue shown in Fig. 1c-d, demonstrating no detected fluorescence due to lipofuscin-derived autofluorescence. **(b)** TRPS analysis by qNano demonstrating the size distribution of exosome particles isolated from AD and control brains. **(c)** Negative staining and electron microscopy shows no structural differences between control and AD brain exosomes. Scale bar, 100 nm. **(d)** InstantBlue protein stain loading control for western blot shown in Fig. 1f. **(e)** Exosomes isolated from dSHSY-5Y control cell conditioned media, was treated with oAβ-AF700 (n=4) or PBS (n=4) and centrifuged at 100,000 x g. No significant increase in AF700 fluorescence was observed, indicating that oAβ not related to the exosomes does not significantly contribute to the detected oAß in the exosome fraction. NS, not significant.

**Supplementary Figure S2.**


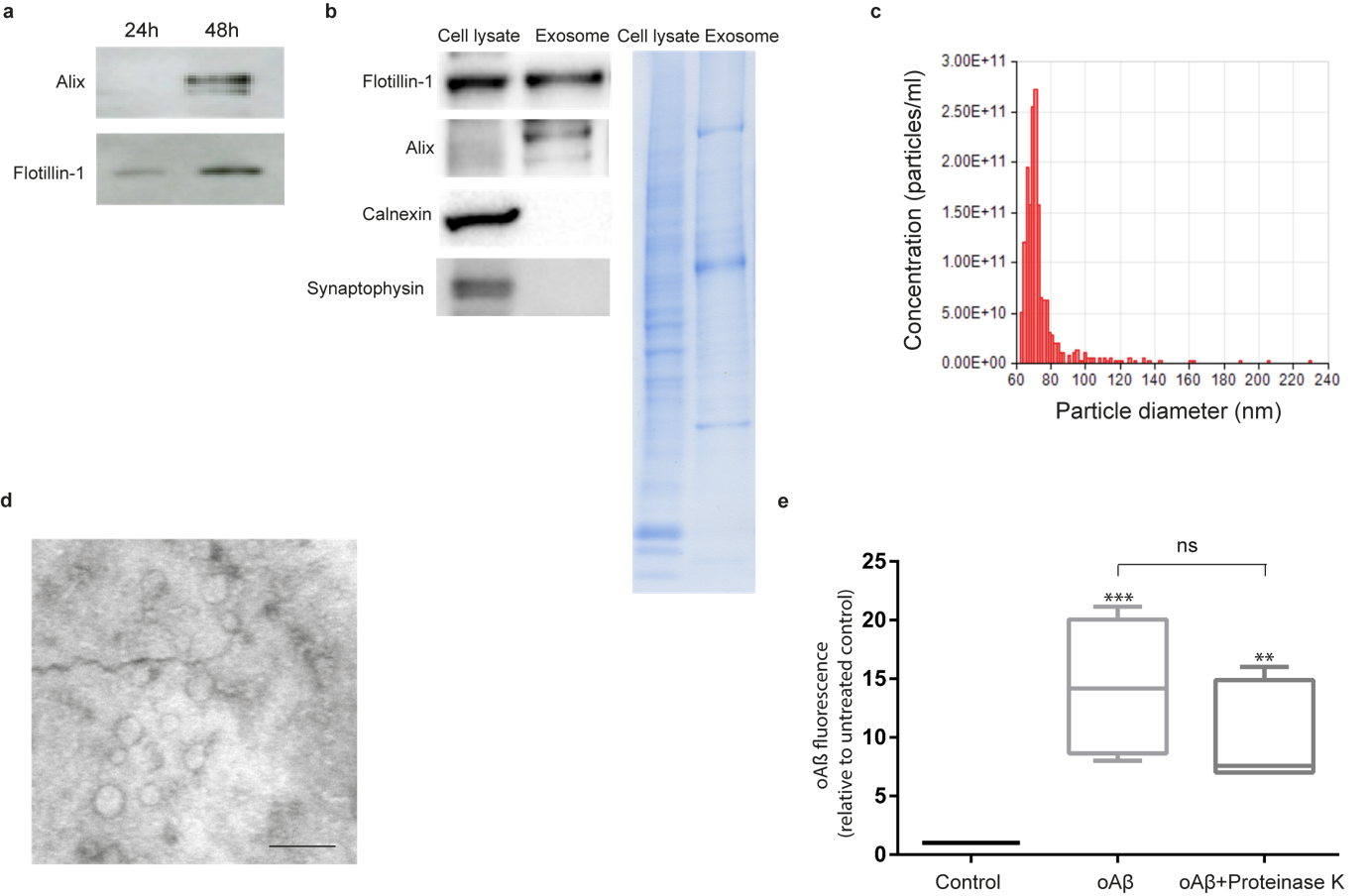


**Supplementary Figure S2. Characterization of exosomes isolated from dSH-SY5Y cells (a)** Immunoblot demonstrating the presence of flotillin1 and Alix in exosomes isolated from 24 and 48 h cultured media of dSHSY-5Y cells. **(b)** Immunoblot showing flotillin-1, alix, calnexin and synaptophysin in exosome and cell lysate, demonstrating no cellular or synapse vesicle contamination in the exosome preparation. InstantBlue protein stain loading control for the western blot is also shown. **(c)** Representative picture of size distribution of exosome sample shows mode diameter 70 nm. **(d)** Electron micrograph of negatively stained exosomes. Scale bar, 200 nm. **(e)** Exosomes were isolated from media of control and oAβ-AF700 treated cells and subjected to proteinase K digestion to confirm the significant intra-vesicular localization of oAβ. Data are represented as the means ± SEM (n = 6); **p < 0.01, ***p < 0.001 by one-way ANOVA with Tukey’s correction.

**Supplementary Figure 3.**


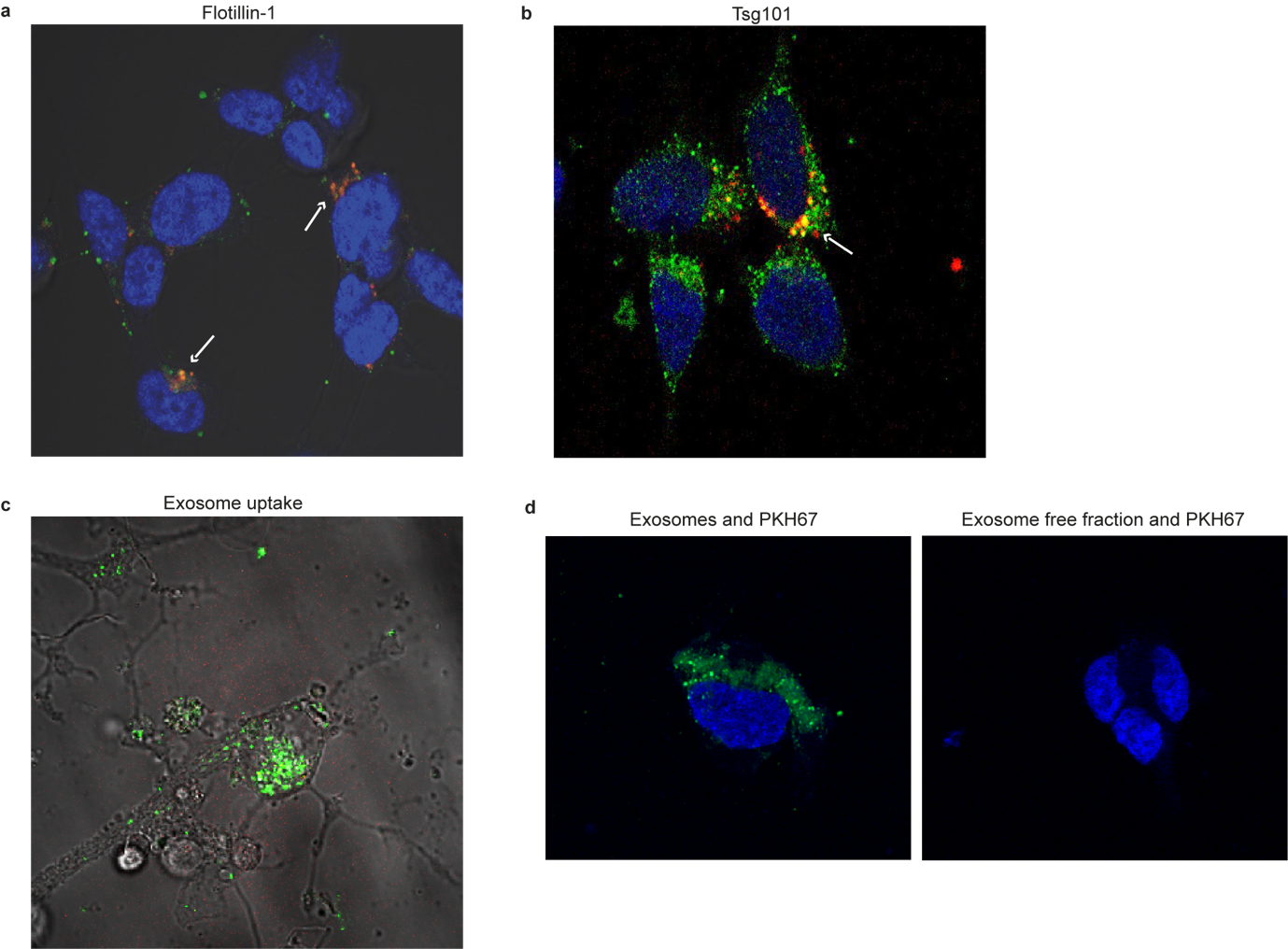


**Supplementary Figure S3. Internalized oAβ co-localizes with exosomes**oAβ-AF700 was added to dSH-SY5Y cells and incubated for 3 hours. After incubation, cells were washed thoroughly with PBS and kept for 24h. After 24 h the cells were fixed and then subjected to immunocytochemistry using Alexa Fluor 488 conjugated secondary antibody (green) showing subcellular targets. Confocal microscopy images show oAβ (red) co-localization (yellow) with **(a)** flotillin-1 (green) and **(b)** TSG101 (green). Arrows indicate co-localization of oAβ with exosomal proteins. **(c)** Exosomes isolated from conditioned media of control cells were taken up by cells and transferred onwards, but no oAβ was detected in these cells. **(d)** Cellular uptake in raSH-SY5Y cells of isolated conditioned media cell exosomes and conditioned media cell exosome free fractions after PKH67 staining showing no PKH67 uptake in the absence of exosomes.

**Supplementary Figure S4.**

**
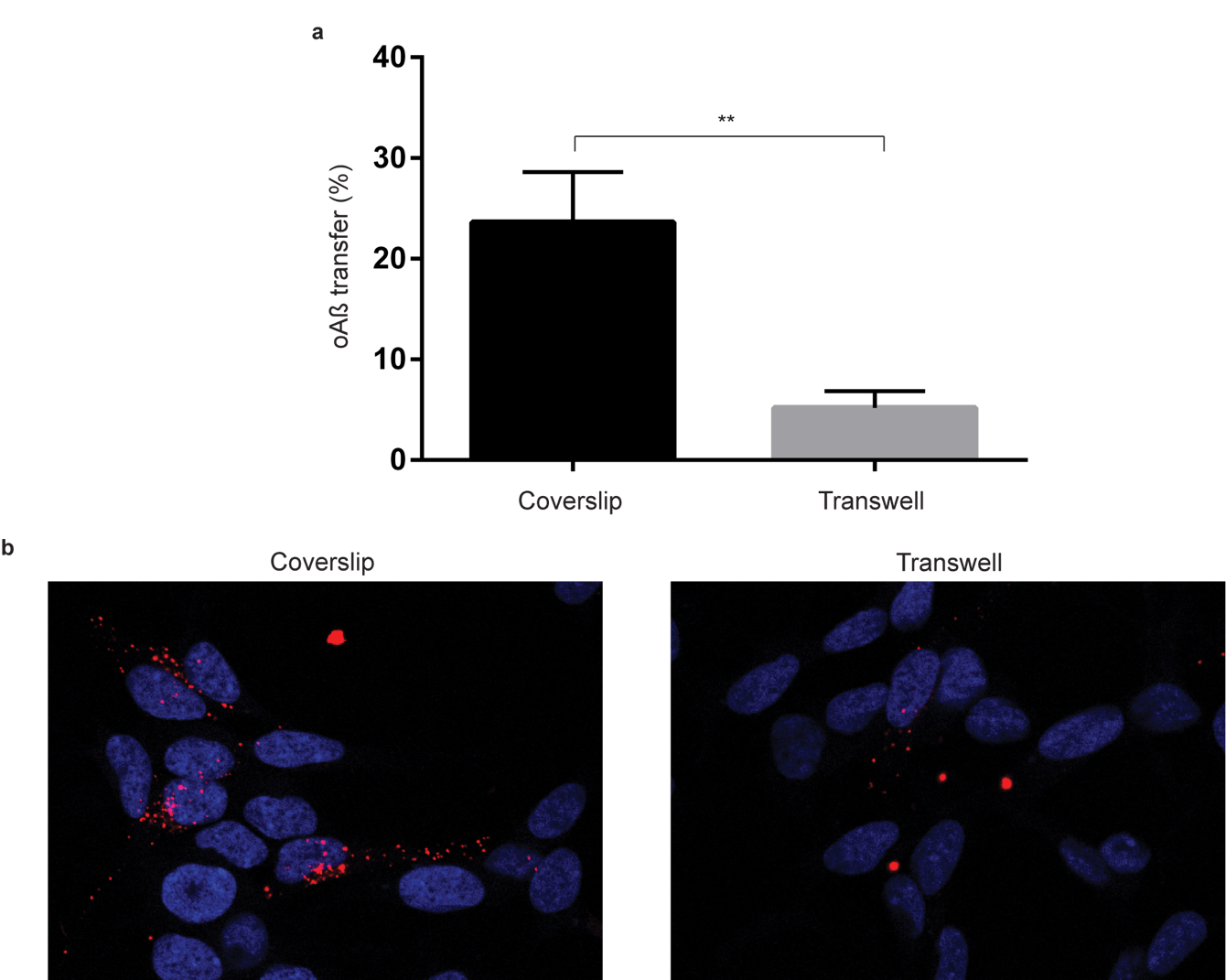
**

**Supplementary Figure S4. oAβ transfer in recipient cells by coverslip and transwell models**oAβ-AF700 or oAβ-TMR was added to donor dSH-SY5Y cells on coverslip. After 3h of incubation at 37°C, donor cells were placed on top of another set of dSH-SY5Y recipient cells (coverslip model) or on top of a transwell polycarbonate membrane filter over recipient cells (transwell model). After 24 h of co-culture, donor cells were removed and the transfer of oAβ to the recipient cells were analyzed by **(a)** flow cytometry showing the proportion of cells containing transfer of oAβ-AF700 or **(b)** visualized by confocal microscopy showing the transfer of oAβ-TMR. Data are presented as mean ± SEM; n = 4; **p < 0.01 by two-tailed unpaired Student’s *t* tests with Welch’s correction.

**Supplementary Figure S5.**

**
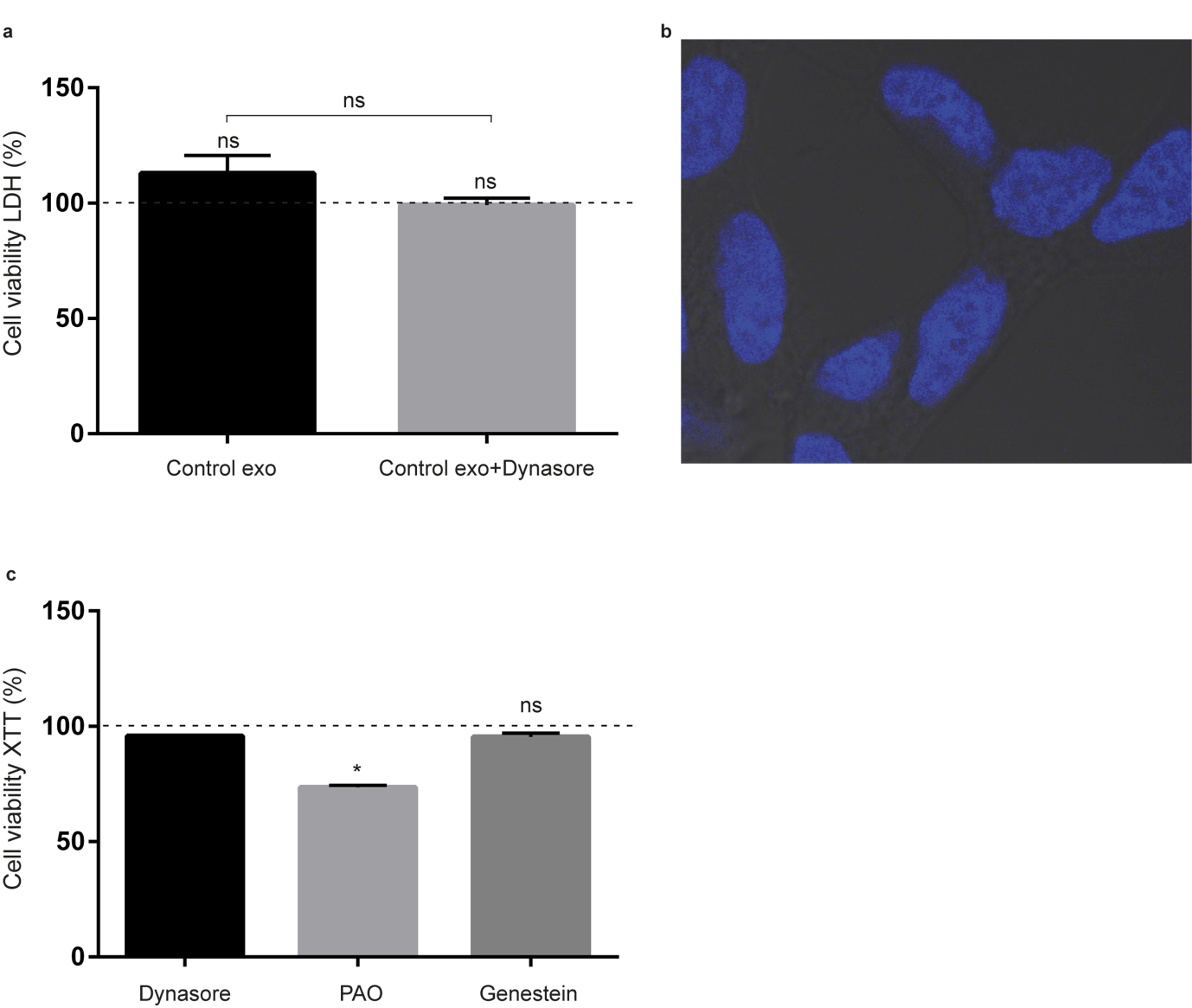
**

**Supplementary Figure S5. Exosome uptake in cells is an active process and non- toxic
(a)** Exosomes isolated from untreated dSH-SY5Y cells has no toxic effect on the recipient cells as assessed by LDH assay. **(b)** dSH-SY5Y were exposed to PKH67 labelled exosomes at 4°C. After 3 h of incubation no fluorescence was found in exposed cells. **(c)** Analysis of cell viability by XTT assay shows no evident cytotoxicity in presence of dynasore or genistein. Data are presented as mean ± SEM; n = 4; *p < 0.05 by two-tailed unpaired Student’s *t* tests with Welch’s correction.

**Supplementary Figure S6.**

**
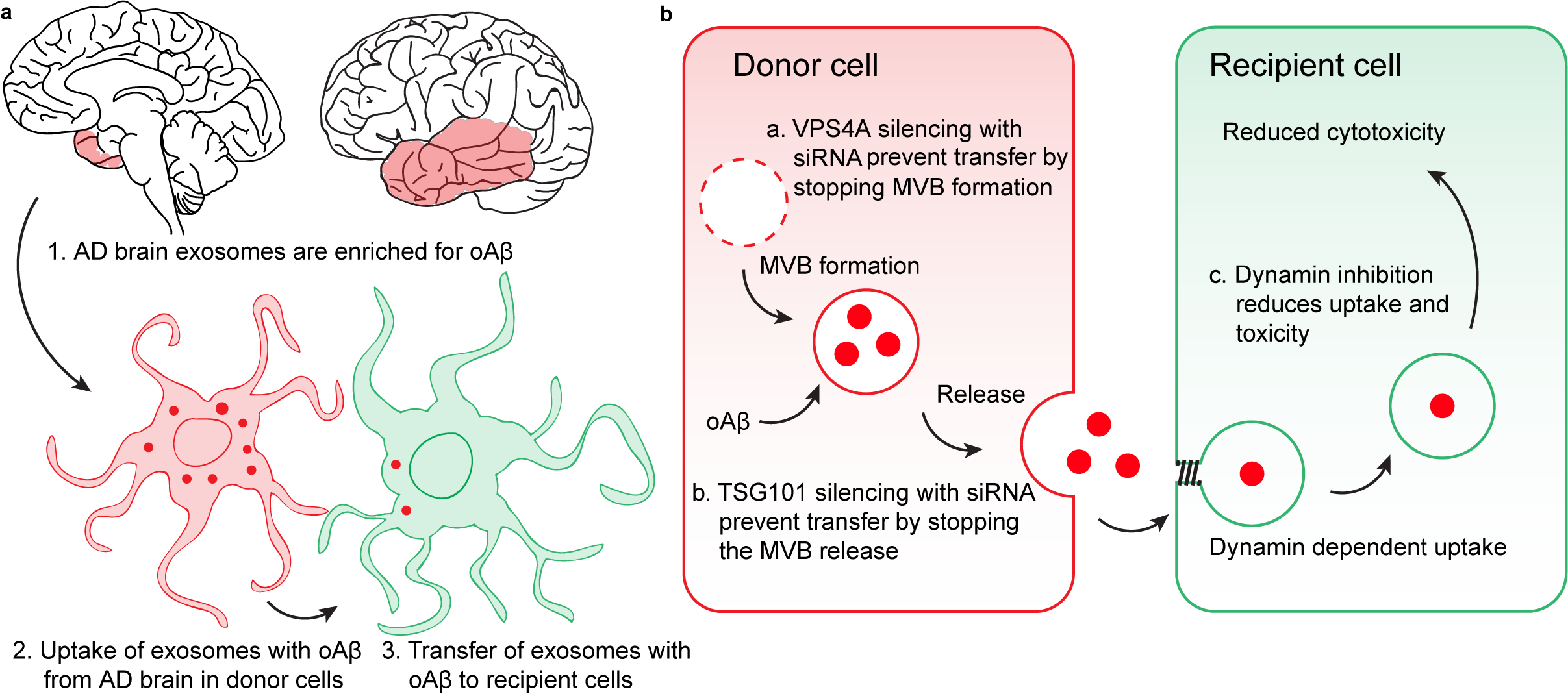
**

**Supplementary Figure S6. Transfer of exosomes containing oAβ between neurons (a)** AD brain exosomes contain increased amount of oAβ (Fig. 1). The exosomes and their oAβ cargo are taken up by neurons, mediating the propagation of oAβ to further neurons (Fig. 2) which causes cellular toxicity (Fig. 3). **(b)** oAβ partially localizes to MVB. If the formation or release of exosomes are inhibited so is the transfer of oAβ (Fig. 4). The transfer can also be reduced by inhibiting the uptake of exosomes (Fig. 5).
